# Single MoO_3_ nanoribbon waveguides: good building blocks as elements and interconnects for nanophotonic applications

**DOI:** 10.1038/srep17388

**Published:** 2015-11-27

**Authors:** Li Zhang, Guoqing Wu, Fuxing Gu, Heping Zeng

**Affiliations:** 1 Shanghai Key Laboratory of Modern Optical System, Engineering Research Center of Optical Instrument and System (Ministry of Education), University of Shanghai for Science and Technology, Shanghai 200093, China; 2 State Key Laboratory of Precision Spectroscopy, East China Normal University, Shanghai, 200062, China

## Abstract

Exploring new nanowaveguide materials and structures is of great scientific interest and technological significance for optical and photonic applications. In this work, high-quality single-crystal MoO_3_ nanoribbons (NRs) are synthesized and used for optical guiding. External light sources are efficiently launched into the single MoO_3_ NRs using silica fiber tapers. It is found that single MoO_3_ NRs are as good nanowaveguides with loss optical losses (typically less than 0.1 dB/μm) and broadband optical guiding in the visible/near-infrared region. Single MoO_3_ NRs have good Raman gains that are comparable to those of semiconductor nanowaveguides, but the second harmonic generation efficiencies are about 4 orders less than those of semiconductor nanowaveguides. And also no any third-order nonlinear optical effects are observed at high pump power. A hybrid Fabry-Pérot cavity containing an active CdSe nanowire and a passive MoO_3_ NR is also demonstrated, and the ability of coupling light from other active nanostructures and fluorescent liquid solutions has been further demonstrated. These optical properties make single MoO_3_ NRs attractive building blocks as elements and interconnects in miniaturized photonic circuitries and devices.

Waveguides are basic building blocks in integrated photonic circuits and devices^1^. The development of ultra-compact and integrated nanophotonic systems represents a challenging direction for exploring phenomena at the nanoscale, and is also expected to play a critical role in future electronic and optoelectronic devices^2^,^3^,^4^,^5^,^6^,^7^. Compared with bulk counterparts, nanowaveguides (e.g., nanowires, nanofibers and nanoribbons) have been constantly gaining interests for investigating light generation, propagation, detection, amplification and modulation, due to their unique advantages such as excellent optical guiding capability, strong light−mater interaction, large evanescent field, and tailorable waveguide dispersion. In the past decades, a variety of materials such as glasses, semiconductors, and polymers have been fabricated for nanowaveguides and used in various applications including photodetectors, chemical and gas sensors, waveguides, microcavity lasers, solar cells and nonlinear optical converters. Therefore, exploring new nanowaveguide materials and structures is of great scientific interest and technological significance for optical and photonic applications.

To become good candidates for waveguides some basic features such as low optical loss and broadband transmission should be matched. Moreover, to ensure delivering light with accurate wavelengths, low activity of nonlinear optical effects in waveguides is also required, because at high-power operation new optical frequencies can be generated via nonlinear optical effects such as second harmonic (SH) generation and stimulated Raman scattering. As a transition metal oxide, molybdenum trioxide (MoO_3_) has gained significant interest in recent years because of its layered structural and functional properties. Various nanostructures of MoO_3_ such as nanosheets, nanorods and nanobelts, have been reported and have found wide applications in electric field emission, energy storage, photocatalysis, and gas sensing^8^,^9^,^10^,^11^,^12^,^13^,^14^,^15^,^16^,^17^. Due to the layered structure, MoO_3_ has also been found to be a good precursor for the synthesis of some important materials such as MoS_2_, and MoSe_2_^18^. Nevertheless, to the best of our knowledge, using MoO_3_ nanostructures as nanowaveguides has not yet been demonstrated. In this work, high-quality single-crystal MoO_3_ nanoribbons (NRs) are synthesized and used for optical guiding. The waveguide behaviors of single MoO_3_ NRs on the dielectric substrates and in the air surrounding conditions are experimentally and theoretically investigated. The results show that MoO_3_ NRs are good nanowaveguides with low optical losses and broadband optical guiding in the visible/near-infrared (VIS/NIR) region, and also have very weak nonlinear optical effects, which make them good building blocks as elements and interconnects for nanophotonic applications.

## Results

### NR synthesis and Characterization

Single-crystal MoO_3_ NRs used here were synthesized by a simple thermal evaporation method, which is carried out in a horizontal quartz tube mounted inside a single-zone furnace^19^,^20^. In the tube, an alumina with MoO_3_ powder (Sinopharm Chemical Reagent Co., Ltd., 99.5% purity,) was placed in the center of the heating zone. Several silicon wafers were placed downstream to collect the deposited MoO_3_ NRs. The carrying gas used was argon with a flow rate of 150 sccm. The evaporation process was carried out at a temperature of 830 °C and a pressure of 300 mTorr. After a growth time of 15 minutes, the temperature was reduced to room temperature and MoO_3_ NRs were grown on the silicon substrates.

**Figure 1:**
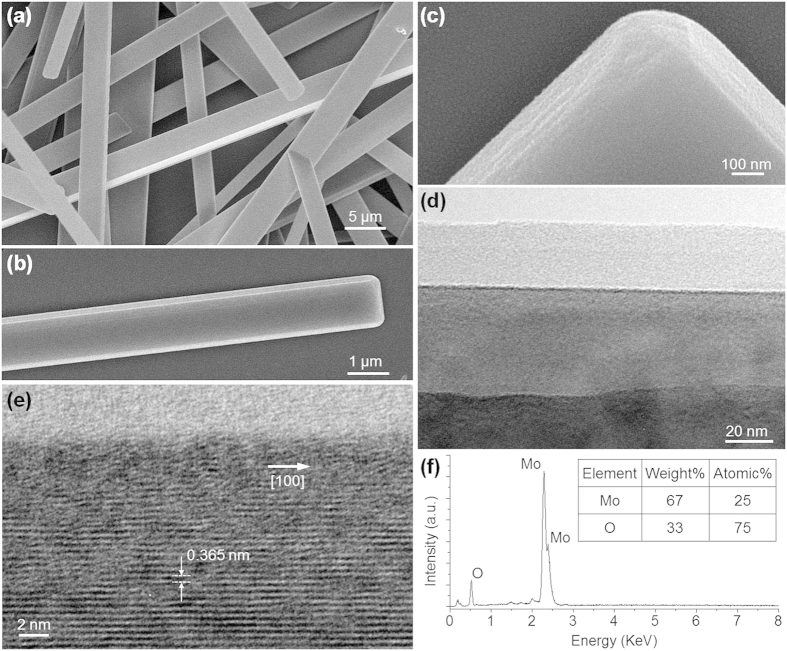
Characterization of as-synthesized MoO_3_ NRs. (**a**) SEM image of as-synthesized MoO_3_ NRs. (**b**) A SEM image of a single MoO_3_ NR and (**c**) an enlarged high-resolution image of its tip. (**d**) TEM image of the layered structure of a MoO_3_ NR. (**e**) High-resolution TEM image of a MoO_3_ NRs, suggesting its single-crystal nature and growing along the [100] direction. (**f**) Energy dispersive spectrometry of as-synthesized MoO_3_ NRs.

A typical scanning electron microscope (SEM) image of as-synthesized NRs is shown in Fig. 1a. The SEM image of a single MoO_3_ NRs in Fig. 1b show the rectangular cross section with smooth surfaces and uniform widths (Fig. 1b), which are favorable for low-loss optical guiding. The enlarged high-resolution SEM image of a tip of the MoO_3_ NR in Fig. 1c and the transmission electron microscope (TEM) image in Fig. 1d clearly show the layered structure of MoO_3_ NRs^15^,^16^. The high-resolution TEM image of one monolayer in Fig. 1e shows their single crystalline nature and the [100] grown direction^12^,^13^,^14^. Energy dispersive spectrometry provided in Fig. 1f reveals that only stoichiometric molybdenum and oxygen signals with a ratio of 1:3 is observed.

### Waveguide behaviors

**Figure 2:**
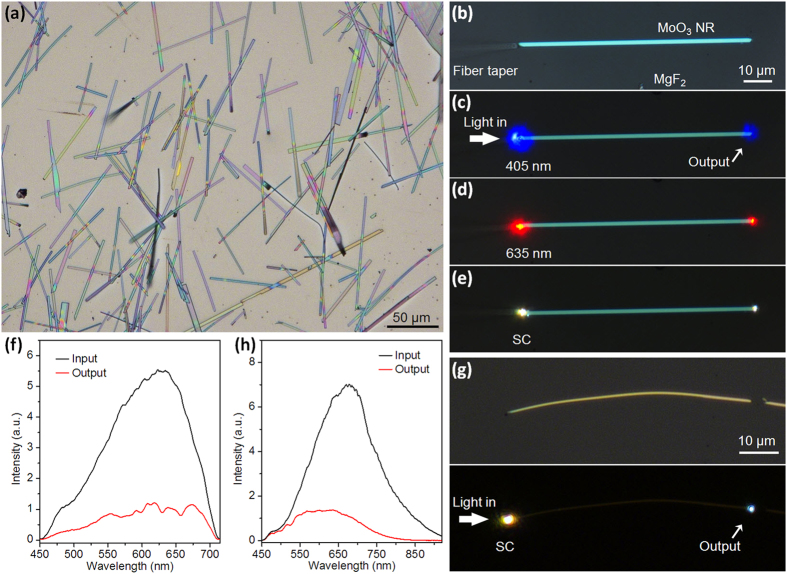
Optical guiding properties of single MoO_3_ NRs on MgF_2_ substrate. (**a**) Optical microscope images of as-synthesized MoO_3_ NRs deposited on an MgF_2_ substrate. (**b**) Light coupling approach in to a 1.65-μm-wide MoO_3_ NR using a fiber taper. (**c**–**e**) Optical microscope images of guiding 405- and 635-nm wavelength lasers, and a broadband SC source into the MoO_3_ NR. (**f**) Spectra collected at the input and output ends of the MoO_3_ NR. (**g**) Optical microscope image of guiding a broadband SC source into a 620-nm-wide MoO_3_ NR and (**h**) spectra collected at its input and output ends.

For optical characterization, as-synthesized MoO_3_ NRs were removed from the growth substrate and deposited on an low refractive-index MgF_2_ substrate (~1.38 around 500 nm), with a typical optical micrograph shown in Fig. 2a. The lengths of MoO_3_ NRs can be as long as hundreds of micrometers. External light is launched into the NRs using a butt-coupling technique^21^,^22^,^23^. In this approach (Fig. 2b) a silica optical fiber taper^20^,^22^ is placed in parallel and contacts with one end of a 1.65-μm-wide MoO_3_ NR, so that the light can be efficiently transferred from the fiber taper to the NR. The fiber taper was mounted on a triple-axis micromanipulator (M-462, Newport) and can be precisely controlled and manipulated under an optical microscope (Nikon 80i) equipped with super-long working distance objectives. Figure 2c,d show optical micrographs of 405- and 635-nm wavelength continuous-wave (CW) lasers coupled into the MoO_3_ NR, respectively. After propagating with a distance of about 80 μm, light is emitted at the distal end of the NR and no scattered spots are observed at the body, suggesting its good optical guiding behavior.

A broadband supercontinuum (SC) source (SuperK Compact, NKT photonics) was also used to characterize the waveguide behaviors. Figure 2e shows an optical micrograph of the NR guiding a SC source, in which two white spots are observed at the input and output ends. Figure 2f shows the spectra at the input and output ends of the NR respectively, both of which are within the visible region and exhibit similar spectral distributions, suggesting that the light in the visible region can be well guided in this NR waveguide. Nevertheless, when MoO_3_ NRs with thin widths are used to guide the SC source, a short-pass filtering effect that longer wavelengths suffer higher losses is observed^24^,^25^. As shown in Fig. 2g in a 620-nm-wide NR, a light spot towards blue color is observed at the output end, because in thinner waveguides the fraction of evanescent waves of longer-wavelength light (Fig. 2h) is higher and induce more energy leaked into the substrate.

**Figure 3:**
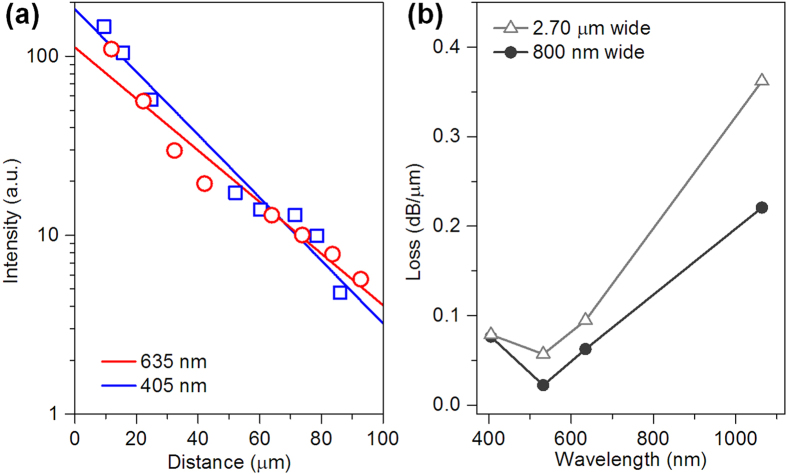
Propagation losses (*α*) in MoO_3_ NRs. (**a**) Propagation distance-dependent output intensity at the wavelengths of 405- and 635 nm respectively, which is measured in a 2.70-μm-wide MoO_3_ NR. (**b**) Wavelength-dependent *α* in MoO_3_ NRs with different widths of 800 nm and 2.70 μm, respectively.

The propagation losses (*α*) of the MoO_3_ NRs on the MgF_2_ substrate were investigated by placing a fiber taper at different locations along the NR waveguides to launching light and measuring the corresponding output intensity (*I*_out_) at the output end of the NR^20^,^26^. Figure 3a plots the measured *I*_out_ at the wavelengths of 405- and 635 nm against varying propagation distances (*x*) in a 2.70-μm-wide MoO_3_ NR. These data points are all well-fitted by a first-order exponential decay function *I*_out_/*I*_in_ = exp(−*αx*)^20^, where *I*_in_ is the input intensity. The obtained *α* are 0.076- and 0.062 dB/μm for the wavelengths of 405- and 635 nm, respectively. Here *α* at 405 nm is higher mainly due to the optical intrinsic absorption that is larger at shorter wavelengths for MoO_3_^27^. In addition, *α* is also highly dependent on the cross-sectional dimensions of the NR waveguides, which is attributed to the leakage of outer evanescent waves that is larger at longer wavelengths^24^,^25^. For reference, Fig. 3b provides *α* obtained in two typical MoO_3_ NRs with different widths of 800 nm and 2.70 μm, respectively. It shows that for both NRs *α* is minimum in the middle spectral region such as around 532 nm, and increases at both the short (due to the strong absorption^27^) and long wavelength regions (due to the leakage of outer evanescent waves),. In the visible region *α* is less than 0.1 dB/μm, and is on the average level compared to those of other dielectric nanowaveguides such as V_2_O_5_ NRs (0.06 dB/μm)^28^. After determine *α*, the coupling efficiencies (*η*) between the fiber taper and the 1.65-μm-wide MoO_3_ NR in Fig. 2b can be roughly calculated by measuring the *I*_out_ and *I*_in_ at the input and output ends. Here *η* is measured as ~48% and 52% for 405- and 635-nm lasers, respectively.

**Figure 4:**
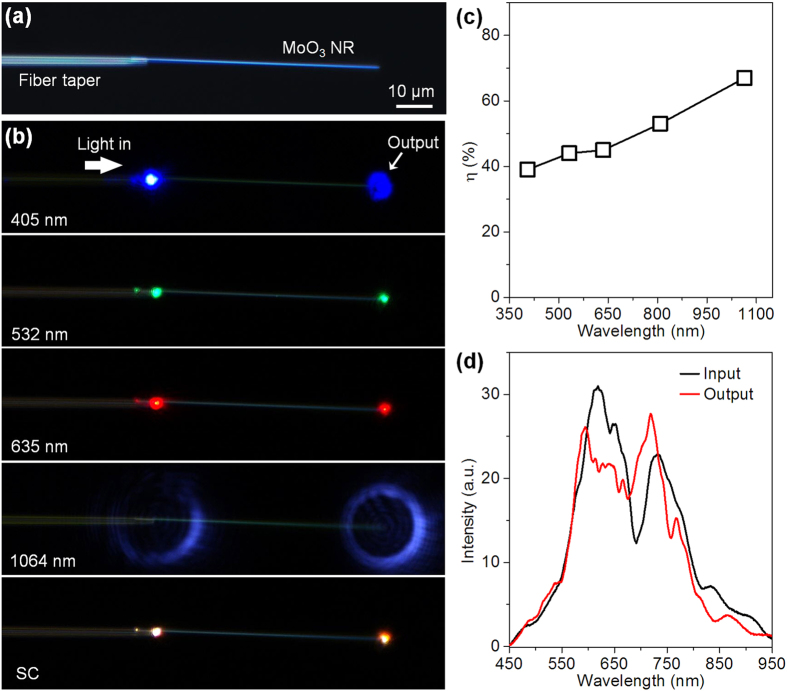
Optical guiding properties of single MoO_3_ NRs in air-surrounding condition. (**a**) Optical microscope image of a single MoO_3_ NRs coupled with a suspended fiber taper, and (**b**) optical microscope images of guiding 405-, 532-, 635-, and 1064-nm wavelength lasers, and a broadband SC source into the MoO_3_ NR. (**c**) Coupling efficiencies (*η*) between the fiber taper and the MoO_3_ NR at different wavelengths. (**d**) Spectra collected at the input and output ends of the MoO_3_ NR.

To enable low-loss optical guiding, the suspension approach^20^,^22^,^24^,^25^ was applicable to MoO_3_ NRs due to its attractive advantage that can significantly reduce the substrate-induced leaking of guided energy. A fiber tapers with sharp tip diameters of less than 300 nm, fabricated from a standard single mode silica optical fiber (SMF-28e, Corning) and mounted by a triple-axis micromanipulator, was used to pick up, transferred, and deposited the MoO_3_ NR onto the target substrates. As shown in Fig. 4a, a 1.12-μm-wide MoO_3_ NR is placed on a tip of a suspended optical microfiber via micromanipulation, and the light can be efficiently coupled into the MoO_3_ NR via an evanescent wave coupling approach^20^,^22^,^24^,^25^. Figure 4b provides optical micrographs of coupling different wavelength lasers and the SC source from a suspended fiber taper into the 1.12-μm-wide MoO_3_ NR. Experiments from dozens of samples show that in the VIS/NIR region the average *α* is about 1 order less than those on the MgF_2_ substrate, i.e., around 0.01 dB/μm. The coupling efficiencies (*η*) between the fiber taper and the MoO_3_ NR are measured as ~39%, 44%, 45%, and 67% for 405-, 532-, 635-, and 1064 nm lasers, respectively (Fig. 4c). For the SC source, a bright light spot is observed at the NR output end, exhibiting a similar white color and spectral intensity (Fig. 4d) as the input one, suggesting high-efficient coupling over 50% in the VIS/NIR region. Also the spectra of the input and the output ends exhibit similar spectral distributions in the VIS/NIR region, suggesting broadband optical guiding capability in the NR waveguide without the filtering effect.

### Investigation of Raman properties

**Figure 5:**
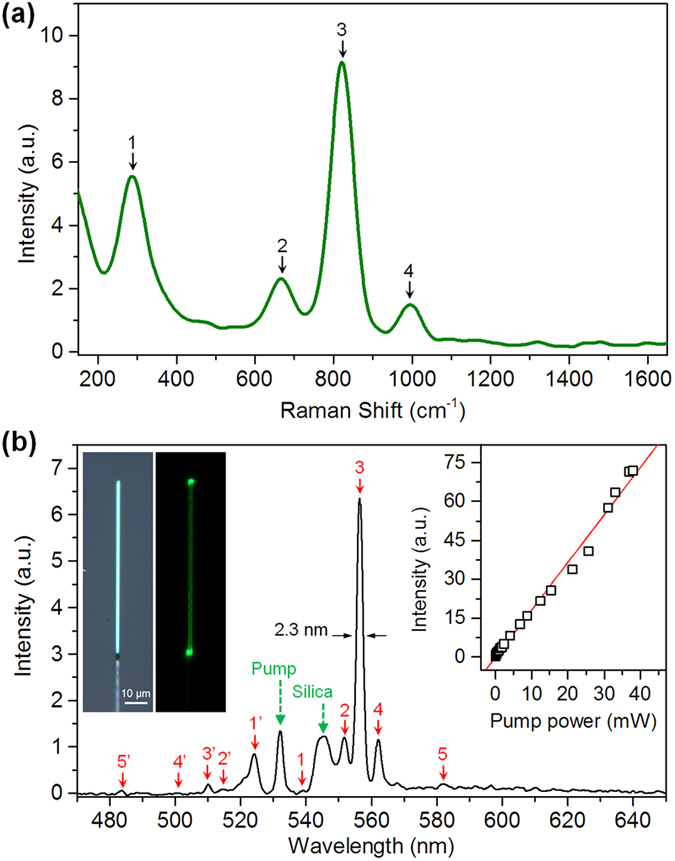
Investigation of Raman properties of single MoO_3_ NRs. (**a**) Typical Raman spectrum of a 1.74-μm-wide MoO_3_ NR with excitation at 532 nm. (**b**) Scattering spectrum of the MoO_3_ NR under pump power of 0.4 mW. Left inset: optical microscope images of the MoO_3_ NRs pumped by a fiber taper, which is captured using an ultra-steep long-pass edge filter. Right inset: pump power-dependent intensity of the 556.4-nm peak.

To figure out the Raman properties of single MoO_3_ NRs, a 532-nm CW monochromatic laser was used as the pump laser and an ultra-steep long-pass edge filter (Semrock LP03-532RE-25) was used to block the pump signals^21^. Figure 5a shows a typical room-temperature Raman spectrum obtained in a 1.74-μm-wide MoO_3_ NR, in which 4 characteristic optical phonon modes are observed. The peak of 1 (287 cm^−1^) corresponds to the wagging mode of double bond O = Mo = O, the peak of 2 (666 cm^−1^) corresponds to the symmetrical stretching vibration of O–Mo–O bonds, the peak of 3 (824 cm^−1^) corresponds to the doubly coordinated oxygen (Mo_2_–O) stretching mode, and the peak of 4 (997 cm^−1^) corresponds to terminal oxygen (Mo^6+^=O) stretching mode^14^,^15^,^17^.

Figure 5b shows a typical scattering spectrum of the 1.74-μm-wide MoO_3_ NR, which was excited with pump power of ~0.4 mW and was collected using a notch filter (Semrock NF01-532U-25). A dominant narrow peak with a wavelength of 556.4 nm and a full width at half-maximum (FWHM) of 2.3 nm is observed, and also several weak peaks lie on both sides of the pump wavelength, corresponding to the Stokes and anti-Stokes scattering waves: (1′) 539.9 and (1) 524.1 nm, (2′) 514.5 and (2) 551.5 nm, (3′) 509.1 and (3) 556.4 nm, and (4′) 500.8 and (4) 562.1 nm. Also two peaks of (5′) 483.6 and (5) 582.1 nm are observed and correspond well to the second-order anti-Stokes and Stokes waves from the 824 cm^−1^ Raman band. The peak around 545 nm is contributed by the silica microfibers. Inset shows that as the pump power increases, the intensity of the dominant 556.4-nm peak exhibits a linear dependence. The measured generation efficiency of Raman emissions at a 0.4-mW pump power is about 10^−6^ mm^−1^, which is comparable to those obtained in semiconductor micro/nanowaveguides (such as CdS nanowires, see Supplementary Fig. S1). As the pump power further increases until the NR was thermally destroyed, the spectral profiles of these Raman signals almost maintained the same as those under low power pump. In addition, no any third-order nonlinear optical effects such as two-photon absorption induced photoluminescence, stimulated Raman scattering, four-wave mixing process and spectral broadening that reported in semiconductor NRs^21^ are observed here.

### Nonlinear optical frequency conversion

**Figure 6:**
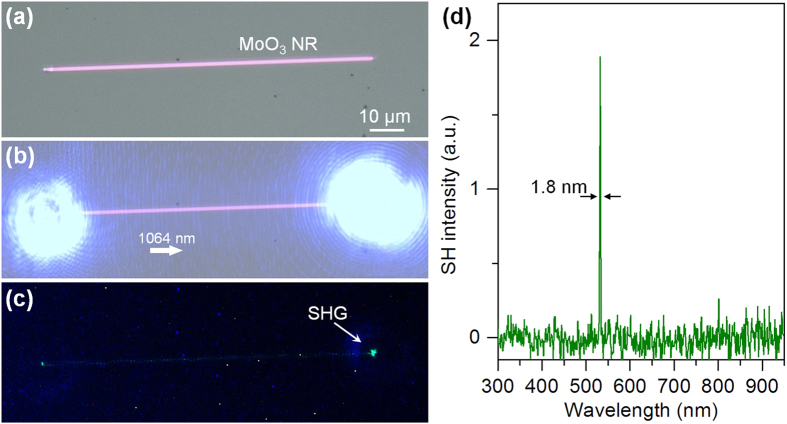
Investigation of SH generation in single MoO_3_ NRs. (**a,b**) optical microscope images of a 960-nm-wide MoO_3_ NRs, which was pumped by launching a 1064-nm CW laser using a fiber taper. The pump power was 50 mW and the optical microscope image (**b**) was captured with an exposure time of 150 s. (**c**) Optical microscope image and (**d**) the spectrum of the generated SH emission in the MoO_3_ NRs.

The property of nonlinear optical frequency conversion in single MoO_3_ NRs is also investigated. A 1064-nm CW laser was launched into a 960-nm-wide and 79-μm-long MoO_3_ NR (Fig. 6a) with a pump power of 50 mW, in which a coupling efficiency about 70% is seen (Fig. 6b). However, a very weak green light spot is observed at the output end of the NR (Fig. 6c). Figure 6(d) shows that the peak wavelength of the green emission is 532 nm with an FWHM of 1.8 nm, corresponding to the SH emission of the 1064-nm fundamental wave. To estimate the generation efficiency from the 1064-nm pump to the 532-nm SH emission, the generated SH intensity was calibrated with a known standard intensity at 532 nm. The estimated efficiency in the 79-μm-long MoO_3_ NR is on the order of 10^−10^ for a pump power of 50 mW, and thus a normalized efficiency is on the order of 10^−8^ mm^−1^. About dozens of MoO_3_ NR samples were tested and all the SH generation efficiencies are around this level; however, this value is at least 4 orders lower than those reported in semiconductor micro/nanowaveguides (such as CdS nanowires, see Supplementary Fig. S2)^23^,^29^. Sum frequency generation was also carried out, but the generation efficiency were as low as that of the SH generation.

### Integration with other nanophotonic structures

**Figure 7:**
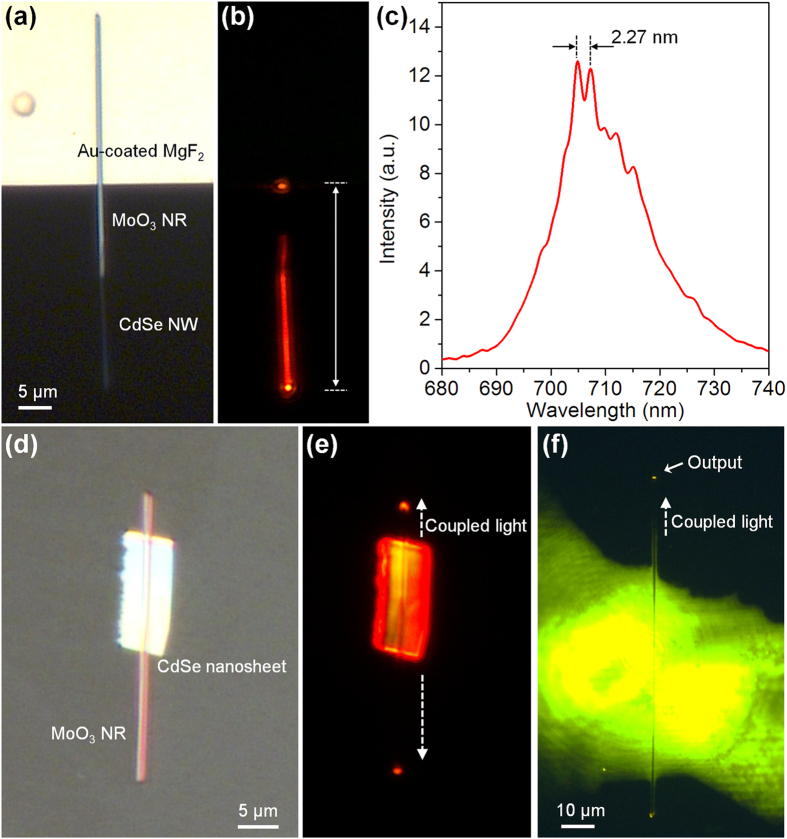
Integration of MoO_3_ NRs with other nanophotonic structures. (**a**) Optical microscope image of the hybrid Fabry-Pérot cavity containing an active CdSe NW and a passive MoO_3_ NR, in which a MoO_3_ NR is placed at the edge of an Au film-coated MgF_2_ substrate, with one tip protrudes out of the substrate, and a CdSe NW is placed parallelly at the tip of the suspended MoO_3_ NR. (**b**) Optical microscope image of the hybrid Fabry-Pérot cavity excited by a 532-nm pulsed pump laser with a power density of 10 W/cm^2^. (**c**) Spectrum of the hybrid cavity collected at the NW–substrate interface. (**d**,**e**) Optical microscope images of coupling light out from a CdSe nanosheet into a MoO_3_ NR. (**f**) Optical microscope image of coupling light out from a small glycol droplet dissolved with 1 mg/mL rhodamine 6 G into a MoO_3_ NR.

To explore their potentials in miniaturized photonic devices and circuitries, firstly a hybrid Fabry-Pérot cavity is demonstrated by coupling single MoO_3_ NRs with active semiconductor nanowires. As shown in Fig. 7a, a MoO_3_ NR is placed at the edge of an Au film-coated MgF_2_ substrate, and one tip of the NR protrudes out of the substrate with a distance of 14 μm; then a CdSe nanowire (NW) is placed parallelly at the tip of the suspended MoO_3_ NR with an overlap of ~5.1 μm. When irradiated by a 532-nm pulsed pump laser (repetition rate: 1 KHz, pulse length: 10 ns) with a power density of 10 W/cm^2^, bright light scattering spots are observed only at the NR right end and the NW–substrate interface. Due to the large optical loss induced by the Au film, no light spots are observed on the MgF_2_ substrate. And also no obvious scattering spots are observed at the coupling area between the NW and NR, because most of the generated photoluminescence from the CdSe NW is efficiently coupled into the MoO_3_ NR. Thus a hybrid Fabry-Pérot cavity containing an active CdSe NW and a passive MoO_3_ NR is constructed. Under a relative high-level pump, a lasing behavior can be generated. Figure 7c shows a typical spectrum collected at the NW–substrate interface under the 10-W/cm^2^ power density. It is observed that the free spectral range (FSR) between the two resonant peaks is 2.27 nm, which is dependent on the relationship^30^,^31^


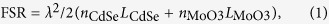


where 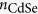 and 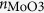 is the group index of the CdSe NW and MoO_3_ NR respectively; 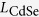 and 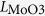 is the effective length of hybrid cavity. Here 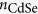 was measured as 5.03 (see Supplementary Fig. S3) and 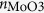 was used as 1.95^27^; 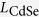 was measured as 17.5 μm and 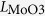 was about 13.4 μm. Thus a calculated FSR is about 2.19 nm, which agrees with the experimental value, suggesting an effective hybrid Fabry-Pérot cavity. By changing the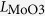, a tunable lasing FSR and lasing peaks may be further achieved.

It is also possible to use MoO_3_ NRs to couple light out from other active nanostructures. Figure 7d shows an optical microscope image of a MoO_3_ NR placed across a CdSe nanosheet. When excited with the 532-nm CW laser, the generated photoluminescence from the nanosheet is coupled into the MoO_3_ NR. Figure 7e clearly shows two bright light spots at the two distal ends. Due to the property of chemical stability, the ability of using MoO_3_ NRs to efficiently couple and guide light in liquid media is also demonstrated. By immersing a MoO_3_ NR in a small glycol droplet dissolved with 1 mg/mL rhodamine 6 G fluorescent dye, Fig. 7f shows that when excited with the 532-nm CW laser, a fraction of the fluorescence is captured by the MoO_3_ NR and guided along to the output ends. Such a waveguide capability is very important for applications such as on-chip chemical and biological sensing and spectroscopy^32^,^33^.

## Discussion

In conclusion, single MoO_3_ NRs have been demonstrated as good nanowaveguides with low optical losses and broadband optical guiding in the visible/near-infrared (VIS/NIR) region. It is found that the optical losses are highly dependent on the intrinsic absorption of MoO_3_ materials and the cross-sectional dimensions of nanowaveguides, and the strong absorption in the ultra-violet spectral range limits their spectral bandwidth. The Raman gains of MoO_3_ NRs are comparable to those of semiconductor nanowaveguides, which can be used as active components for device applications. The SH generation efficiency is about 4 orders less than those of semiconductor nanowaveguides, and also no any third-order nonlinear optical effects are observed in MoO_3_ NRs at high-level pump power. To explore their potentials in miniaturized photonic circuitry and devices, a hybrid Fabry-Pérot cavity containing an active CdSe NW and a passive MoO_3_ NR is demonstrated. The ability of coupling light from other active nanostructures such as semiconductor nanosheets and fluorescent liquid solutions has been further demonstrated. With these optical properties single MoO_3_ NRs can deliver light signals accurately as elements and interconnects such as couplers, resonators, and interferometers, and may provide diverse flexibility to carry out complicated tasks in integrated optical and photonic micro/nano-systems. Furthermore, since MoO_3_ NRs have single-crystal layered structures our results may also open opportunity to study the mono- or few-layered nanostructures using an optical guiding approach.

## Additional Information

**How to cite this article**: Zhang, L. *et al.* Single MoO_3_ nanoribbon waveguides: good building blocks as elements and interconnects for nanophotonic applications. *Sci. Rep.* **5**, 17388; doi: 10.1038/srep17388 (2015).

## Supplementary Material

Supplementary Information
