# Supplementary material for: Single MoO3 nanoribbon waveguides: good building blocks as elements and interconnects for nanophotonic applications
Source: Sci Rep. 2015 Nov 27;5:17388. doi: 10.1038/srep17388 (PMC4661722; doi:10.1038/srep17388)
Supplement: Supplementary Information [file srep17388-s1.pdf]

Supplementary Information

Single MoO_3 nanoribbon waveguides: good building blocks as elements and interconnects for nanophotonic applications

Li Zhang[1], Guoqing Wu[1], Fuxing Gu[1]* & Heping Zeng[1,2,]*

[1]Shanghai Key Laboratory of Modern Optical System, Engineering Research Center of Optical Instrument and System (Ministry of Education), University of Shanghai for Science and Technology, Shanghai 200093, China

[2]State Key Laboratory of Precision Spectroscopy, East China Normal University, Shanghai, 200062, China

Correspondence and requests for materials should be addressed to F.Gu. (email: fuxinggu@gmail.com) and H.Zeng (hpzeng@phy.ecnu.edu.cn)

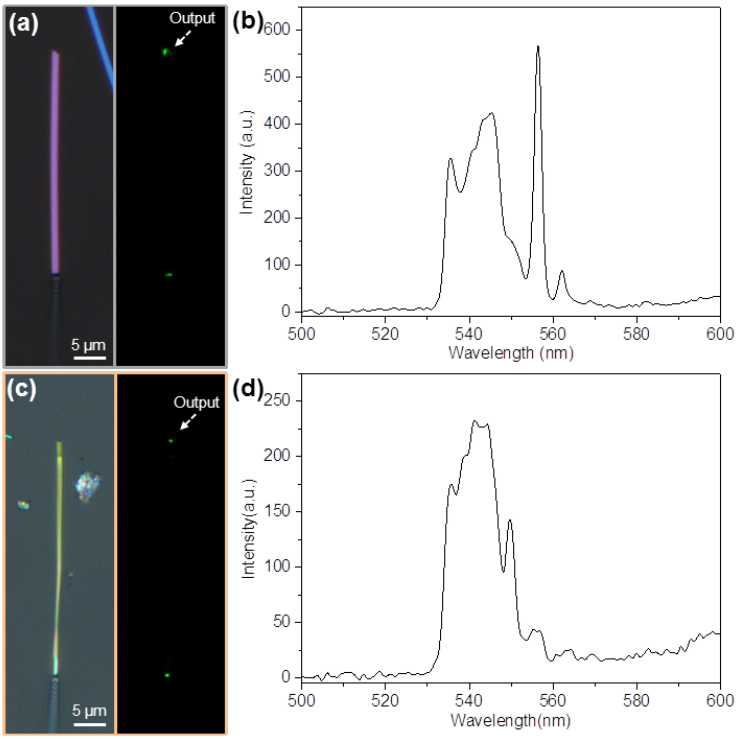

Figure S1| Comparison of Raman properties between single MoO_3 NRs and single CdS NWs. (a) Optical microscope images and (b) spectrum of a single MoO_3 NR. (c) Optical microscope images and (d) spectrum of a single CdS NW. These results are obtained with a 532-nm CW laser under a pump power of 390 μW. An ultra-steep long-pass edge filter (Semrock LP03-532RE-25) was used to filter the pump laser. These optical microscope images were all captured with an exposure time of 200 ms. From the optical microscope images and spectra, it is found that the Raman intensity of the MoO_3 NR is comparable to that of the CdS NW.

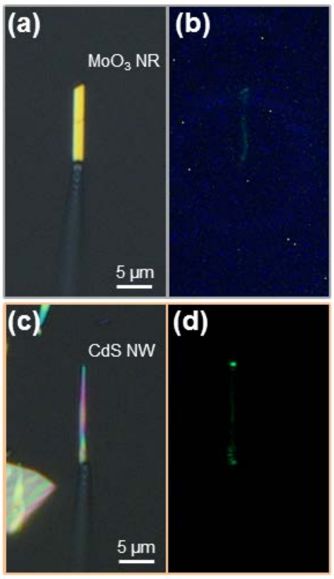

Figure S2| Comparison of second harmonic (SH) generation between single MoO_3 NRs and single CdS NWs. Optical microscope images of (a) a single MoO_3 NR and (b) the generated SH emission. Optical microscope

images of (a) a single CdS NW and (b) the generated SH emission. These results are obtained with a 1064-nm CW laser under a pump power of 50 mW. An ultra-steep long-pass edge filter (Semrock LP03-532RE-25) was used to filter the pump laser. The optical microscope image (b) is captured with an exposure time of 150 s, and the image (d) is captured with an exposure time of 40 ms. By measuring the pixels in the optical microscope images, it is found that the SH generation efficiency of the MoO_3 NR is at least 4 orders lower than those in the CdS NW.

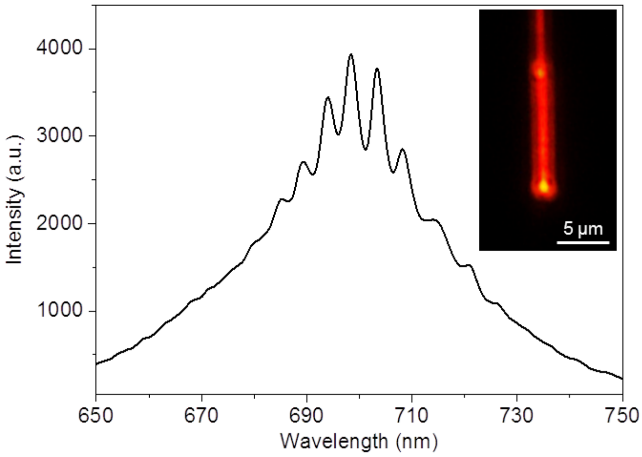

Figure S3| Calculation of group index (n_{CdSe}) of CdSe NW. To obtain the n_{CdSe}, the CdSe NW was placed on a tip of a suspended silica microfiber, and then was excited using a 532-nm pulsed laser (repetition rate: 1 KHz, pulse length: 10 ns). Under a pump power of around 0.5 mW, a resonant lasing action around a wavelength of 703.2 nm was observed. The FSR is measured as 5.28 nm, and L_{CdSe} is measured as 9.3 μm. By using the repationship:

$$\mathrm{FSR}=\lambda^2 / 2n_{CdSe}L_{CdSe}, \qquad (1)$$

thus the n_{CdSe} can be obtained as 5.03.
